# Recent Advances in Screening of Anti-*Campylobacter* Activity in Probiotics for Use in Poultry

**DOI:** 10.3389/fmicb.2016.00553

**Published:** 2016-05-31

**Authors:** Manuel J. Saint-Cyr, Muriel Guyard-Nicodème, Soumaya Messaoudi, Marianne Chemaly, Jean-Michel Cappelier, Xavier Dousset, Nabila Haddad

**Affiliations:** ^1^SECALIM Unit UMR1014, Oniris, INRA, Université Bretagne LoireNantes, France; ^2^Hygiene and Quality of Poultry and Pork Products Unit, Ploufragan/Plouzané Laboratory, ANSES, Université Bretagne LoirePloufragan, France

**Keywords:** *Campylobacter*, poultry, probiotics, screening, *in vitro* virulence, *in vivo* colonization

## Abstract

Campylobacteriosis is the most common cause of bacterial gastroenteritis worldwide. *Campylobacter* species involved in this infection usually include the thermotolerant species *Campylobacter jejuni*. The major reservoir for *C. jejuni* leading to human infections is commercial broiler chickens. Poultry flocks are frequently colonized by *C. jejuni* without any apparent symptoms. Risk assessment analyses have identified the handling and consumption of poultry meat as one of the most important sources of human campylobacteriosis, so elimination of *Campylobacter* in the poultry reservoir is a crucial step in the control of this foodborne infection. To date, the use of probiotics has demonstrated promising results to reduce *Campylobacter* colonization. This review provides recent insights into methods used for probiotic screening to reduce the prevalence and colonization of *Campylobacter* at the farm level. Different eukaryotic epithelial cell lines are employed to screen probiotics with an anti-*Campylobacter* activity and yield useful information about the inhibition mechanism involved. These *in vitro* virulence models involve only human intestinal or cervical cell lines whereas the use of avian cell lines could be a preliminary step to investigate mechanisms of *C. jejuni* colonization in poultry in the presence of probiotics. In addition, *in vivo* trials to evaluate the effect of probiotics on *Campylobacter* colonization are conducted, taking into account the complexity introduced by the host, the feed, and the microbiota. However, the heterogeneity of the protocols used and the short time duration of the experiments lead to results that are difficult to compare and draw conclusions at the slaughter-age of broilers. Nevertheless, the combined approach using complementary *in vitro* and *in vivo* tools (cell cultures and animal experiments) leads to a better characterization of probiotic strains and could be employed to assess reduced *Campylobacter* spp. colonization in chickens if some parameters are optimized.

## Introduction

Food safety is of fundamental importance to the consumer, the food industry and the economy. The incidence of foodborne diseases is still increasing in the European Union (EU) (Hugas et al., 2009; EFSA, 2015), mainly caused by the presence and/or the growth of pathogenic bacteria in food. *Campylobacter* and *Salmonella* are among the leading causes of bacterial foodborne illness and are therefore considered as major public health concern (Scallan et al., 2011). In many countries, the number of human campylobacteriosis cases has considerably increased to exceed the number of *Salmonella* infections in humans by 2–3-fold (EFSA, 2010). The disease is characterized by watery or bloody diarrhea, abdominal cramps and nausea (Blaser et al., 2008). Post-infection complications include peripheral neuropathies, Guillain-Barré and Miller Fisher syndromes, and functional bowel diseases, such as irritable bowel syndrome (Moore et al., 2005). Hospitalization occurs in 10% of cases (Bessell et al., 2010) and 0.2% end in death (Adak et al., 2005). In 2013, with 214,779 confirmed cases corresponding to a notification rate of 64.8 cases per 100,000 inhabitants, campylobacteriosis was the most frequently reported zoonotic disease in humans in the EU (EFSA, 2015). There are several species of *Campylobacter* (*C*. *jejuni, C. coli, C. lari*, and *C. upsaliensis*) capable of causing human illness. However, *C. jejuni* is the one most frequently involved in zoonotic infections (Hugas et al., 2009). It is believed to be responsible for 400–500 million cases of gastroenteritis worldwide per year (Olson et al., 2008). *Campylobacter* cases are often associated with very large costs, i.e., medical expenses, lost wages, legal costs, and other indirect expenses. Only sporadic data are available on the overall costs of *Campylobacter* infections but campylobacteriosis and its sequelae in the EU are calculated to cost 0.35 million disability-adjusted life-years per year, totaling 2.4 billion per year (EFSA, 2014). Annual costs for the US were calculated to range between 1.2 and 4 billion $ (Batz et al., 2012; Eberle and Kiess, 2012). Batz et al. (2014) estimated 16 QALY (quality-adjusted life years) lost per 1000 campylobacteriosis cases; with more than 828,500 cases annually reported, global estimation is around 13,256 QALY losses in the US per year. More recently, Scharff (2015) gives a QALY analysis for all foodborne pathogens including *Campylobacter*.

*Campylobacter* is a commensal organism routinely found in cattle, sheep, swine, and avian species, the latter being the most common host. Numerous studies have already emphasized the importance of poultry as a reservoir of *Campylobacter* (Herman et al., 2003; Hermans et al., 2012; Sasaki et al., 2013) and epidemiological evidence indicates poultry and poultry products are a significant source of human infection (Mor-Mur and Yuste, 2010; EFSA, 2011). In particular, broiler meat is considered the main foodborne source of *Campylobacter* human infection (Nadeau et al., 2003; Nielsen et al., 2006; Silva et al., 2011; EFSA, 2014). Recently, a national prospective case-control study of factors associated with *Campylobacter* infection confirmed that consumption of poultry remains an important exposure for campylobacteriosis in Norway (MacDonald et al., 2015). Good hygiene and biosecurity practices have been implemented to avoid, or at least, reduce contamination (Gibbens et al., 2001) but are considered as not sufficient (Hermans et al., 2011). Considering this information, it is imperative to find a way to minimize *Campylobacter* presence at the farm level in order to reduce the risk of transmission throughout the processing stages. Reducing the proportion of *Campylobacter*-infected poultry flocks and/or reducing the number of *Campylobacter* in live poultry will considerably lower the risk to consumers (Keener et al., 2004; Westrell et al., 2009). Furthermore, prevention of disease in humans and a reduction in the pathogen reservoir in farm animals, without the need for antibiotics, are of both ecological and financial benefit to society.

Regarding the emergence of antibiotic resistance in livestock breeding (Schwarz and Chaslus-Dancla, 2001), poultry farmers turned to new solutions to maintain animal welfare without affecting performance parameters. Over the past years, researchers are considering the use of probiotics as feed additives in poultry nutrition (Kabir, 2009). Probiotics are usually defined as “live microorganisms which, when administered in adequate amounts, confer a health benefit on the host” (Hill et al., 2014). In 2002, the United Nations FAO/WHO Working Group generated new guidelines for the development and evaluation of probiotics found in foods (Reid, 2005). They are acceptable and cost-effective alternatives to antibiotics.

This review provides recent insights into the technological and scientific advances to reduce the prevalence and colonization of *Campylobacter* at the farm level with an emphasis on the screening of probiotics.

## *Campylobacter* in poultry

The prevalence of *Campylobacter* spp. in broiler chicken batches varies considerably between EU countries; in 2008, it ranged from 2 to 100% (average of 71%) (EFSA, 2010). In France, *Campylobacter* is present at all stages of the food chain with a very high prevalence of infection: 70–100% of broiler chicken batches on their arrival at the slaughterhouse (Hue et al., 2010); 72–77% of individual cecal portage during rearing and on arrival at the slaughterhouse; 88% of carcasses and 76% of products at the retail level (Chemaly et al., 2012; Guyard-Nicodème et al., 2015). These results for France are broadly comparable to some high-prevalence countries in the EU. Epidemiological studies have identified potential risk factors associated with *Campylobacter* colonization of flocks (Refrégier-Petton et al., 2001; Bull et al., 2006; Allain et al., 2014; Robyn et al., 2015), including season (Huneau-Salaün et al., 2007), drinking water quality (Ellis-Iversen et al., 2009), or lack of hygienic barriers (Huneau-Salaün et al., 2007).

Colonization of broiler flocks with *Campylobacter* species typically occurs between 2 and 3 weeks of age (Newell et al., 2011). The infection is mostly asymptomatic although chickens can harbor very high levels of *Campylobacter* in the gut, from 5 to 9 log_10_ CFU/g of cecal content (Saleha, 2002; Hansson et al., 2010). Once in a flock, *Campylobacter* is rapidly transmitted between birds by the fecal-oral route (Wassenaar, 2011) and *Campylobacter*-positive birds often remain colonized until slaughter (Newell et al., 2011). During transport of birds (Hansson et al., 2005) and carcass dressing, the surface of broiler carcasses and the plant environment are contaminated by fecal material from the gastrointestinal tract (Herman et al., 2003; Rasschaert et al., 2006; Rosenquist et al., 2006; Reich et al., 2008). Contamination of carcasses with *Campylobacter* occurs mainly during defeathering, evisceration and chilling operations (Sánchez et al., 2002; Stern and Robach, 2003; Takahashi et al., 2006). The bacteria can thus survive during poultry processing through to human consumption, causing subsequent illness as demonstrated by a Danish prospective case-control study (MacDonald et al., 2015). Reducing the cecal *Campylobacter* load in poultry during primary production is expected to decrease significantly the contamination levels of the carcasses of colonized animals after processing, and to reduce the incidence of human campylobacteriosis (Lin, 2009; Hermans et al., 2012).

## *Campylobacter* control at farm level

A possible way to reduce *Campylobacter* contamination in poultry is by actions at the primary production level. To date, three general strategies have been proposed to control *Campylobacter* in poultry at the farm level: (i) a reduction in environmental exposure (Van de Giessen et al., 1998), (ii) an increase in the poultry host's resistance to reduce *Campylobacter* carriage in the gut (Neal-McKinney et al., 2014), and (iii) the use of antimicrobial alternatives to reduce and even eliminate *Campylobacter* from colonized chickens (Ghareeb et al., 2012).

Preventive strategy consists in the application of generic control measures that have an impact on transmission routes of pathogens; and therefore may reduce *Campylobacter* level in poultry. This includes in particular biosecurity, good husbandry as well as hygiene measures. Biosecurity practices at the farm have been reviewed by Newell et al. (2011) and include disinfecting poultry houses, boot dips (Galanis, 2007), fly screens (Hald et al., 2007), disinfecting equipment and vehicles, and treating the flock water supply (Wassenaar, 2011). Nevertheless, contamination is only reduced at the farm level while *Campylobacter* remains widespread in the outside environment, for example in other animal reservoirs (Devane et al., 2005). Once the flock was infected by *Campylobacter*, biosecurity measures became useless. Therefore, additional actions are necessary to fight this foodborne pathogen (Hermans et al., 2011; Robyn et al., 2015), such as vaccination, bacteriocin treatment, or probiotics.

### Strategies in progress

Complementary practices currently being investigated (Table 1) include vaccination (De Zoete et al., 2007; Meunier et al., 2016b), bacteriocins (Svetoch and Stern, 2010; Messaoudi et al., 2012a), bacteriophages (Monk et al., 2010), prebiotics (Gaggìa et al., 2010), and probiotics (Kergourlay et al., 2012; Messaoudi et al., 2012b, 2013). To date, there are still no effective and consistent immune interventions, primarily due to the lack of understanding of the protective immunity, the antigenic variability of different *Campylobacter* strains, and the inability of current vaccination to induce a strong and persistent mucosal immune response in chickens (Meunier et al., 2016a). Studies using bacteriophages showed that they were partly and temporally effective in reducing *Campylobacter* in broilers. This could be explained by the fact that *Campylobacter* develop resistance to bacteriophages (Janež and Loc-Carrillo, 2013) and that these may be strain-specific and only effective against certain *Campylobacter* strains (Loc-Carrillo et al., 2005).

**Table 1:** **Strategies in progress to control *Campylobacter* at the farm level**.

**Strategy**	**Principle**	**Advantage**	**Drawback**
Vaccination	Improvement of the immune response against *Campylobacter*	Easy to use	Antigenic variability of *Campylobacter* strains
Bacteriophage therapy	Use of specific bacterial virus to kill *Campylobacter*	Rapid action	Selection of resistant *Campylobacter* strains Production cost Diversity of *Campylobacter* strains
Bacteriocin treatment	Use of bacteria-produced antimicrobial compounds against *Campylobacter*	Easy to use	Production cost Variable sensitivity of *Campylobacter* strains
Prebiotics	Incorporation of feed additives to improve beneficial avian gut microbiota	Easy to use Production cost	Dependence on the avian gut microbiota
Probiotics	Administration of beneficial microorganisms with anti-*Campylobacter* activity	Easy to produce and to use Production cost Mix of multiple species Different ways of inhibiting *Campylobacter*	Variable sensitivity of *Campylobacter* strains

Interestingly, prebiotics and bacteriocins can be used together to probiotics to potentially increase the anti-*Campylobacter* activity. Prebiotics are non-digestible ingredients, such as fructo-oligosaccharides (Patterson and Burkholder, 2003), which enhance the growth of gut commensal bacteria that have probiotic properties, i.e., *Bifidobacterium* (*Bf*.) and *Lactobacillus* (*Lb*.) (Roberfroid, 1998), while bacteriocins are ribosomally-synthesized antimicrobial peptides produced by bacteria. Few studies have been conducted to evaluate the efficacy of prebiotics in reducing *Campylobacter* colonization in poultry. The addition of mannanoligosaccharide to the feed of naturally-infected birds and xylanase to artificially-infected broilers resulted in a statistically significant decrease of 0.3 log in cecal *C. jejuni* counts (Baurhoo et al., 2009). Concerning bacteriocins, for example, Messaoudi et al. (2012a) showed that the viable population of *C. jejuni* NCTC 11168 pure cultures decreased by 2 log when growth was performed in the presence of salivaricin SMXD51. Administration of enterocin E-760-treated feed significantly reduced the colonization of young broiler chicks experimentally challenged and colonized with two strains of *C. jejuni* by more than 8 log CFU (Line et al., 2008). Another *in vivo* study on chickens infected with *C. jejuni* and *Salmonella enteritidis*, demonstrated that treatment with L-1077, the bacteriocin produced by *Lb. salivarius* NRRL B-50053, reduced by more than 4 log the number of bacteria per gram of cecal content (Svetoch et al., 2011). Majority of the bacterial antimicrobial peptides active against *C. jejuni* were isolated from *Bacillus* and *Paenibacillus* spp., and from the lactic acid bacteria (Lohans et al., 2015). Svetoch and Stern (2010) have reviewed bacteriocin applications to reduce the cecal *Campylobacter* counts in broiler chickens of colonized flocks. This strategy is of limited relevance for the moment because purity and yields of bacteriocins, after purification, are low. This could be due, in part, to their low molecular weight and to the design of the purification processes employed so far (Carolissen-Mackay et al., 1997). In addition, hydrophobic peptides are often only produced in small amounts (Berjeaud and Cenatiempo, 2004). But efforts are underway and current strategies to enhance yield of bacteriocins were recently described by Zacharof (2015).

**Figure 1:**
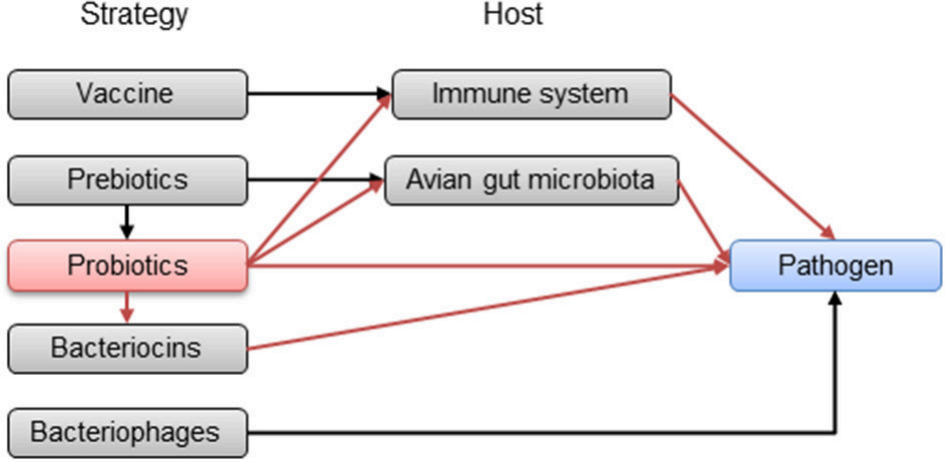
**Potential pathways of the strategies in progress to reduce avian gut pathogens in poultry**. Red arrows represent probiotic pathways.

Prohibition of antibiotics in poultry feed in Europe and the problems inherent in developing new vaccines make probiotics a promising prophylactic alternative to control *C. jejuni* in broiler chickens during rearing at the farm level (Table 1). They could act in multiple ways, at the same time, against pathogens in contrast to other more specific strategies (vaccination or bacteriophages) (Figure 1). In fact, probiotics are already used in the poultry industry for preventing or reducing the occurrence of *Salmonella* infection in poultry and for enhancing the growth performance of broiler chickens (Tellez et al., 2013). Their impact on poultry nutrition is of great importance for the proper utilization of nutrients.

### Probiotics: Attractive and natural antimicrobial agents

Being living microorganisms, probiotics can stimulate gut microbiota which contributes to keep the host healthy (Fuller, 1989; Sanders, 2011). Based on *in vitro* assays, these modifications include stimulation of the immune system (Smits et al., 2005), acidification of the environment (Ogawa et al., 2001), secretion of active metabolites against pathogens, such as bacteriocins (Marciňáková et al., 2004) or hydrogen peroxide (Batdorj et al., 2007), and competition with the pathogens for nutrients or sites for adherence on the mucous membrane or the host epithelial cells (Bernet et al., 1994). These abilities can be useful to control pathogen infection and probiotic treatment has been linked with beneficial effects against gastrointestinal pathogens using animal models. For example, a mixture of *Lactobacillus* spp. strains reduced gastric inflammation and bacterial colonization in *Helicobacter pylori*-infected mice (Johnson-Henry et al., 2004). A five-strain probiotic combination (two strains of *Lb. murinus* and one strain each of *Lb. salivarius, Lb. pentosus*, and *Pediococcus pentosaceous*) reduced pathogen shedding and alleviated disease signs in pigs challenged with *S*. *enterica* serovar Typhimurium (Casey et al., 2007). Pascual et al. (1999) showed that a treatment with *Lb. salivarius* CTC2197 prevented *S. enterica* serovar Enteritidis colonization in chickens. In addition, some probiotic strains as feed supplements can also prevent gastrointestinal infection in broiler chickens (Tellez et al., 2001).

**Figure 2:**
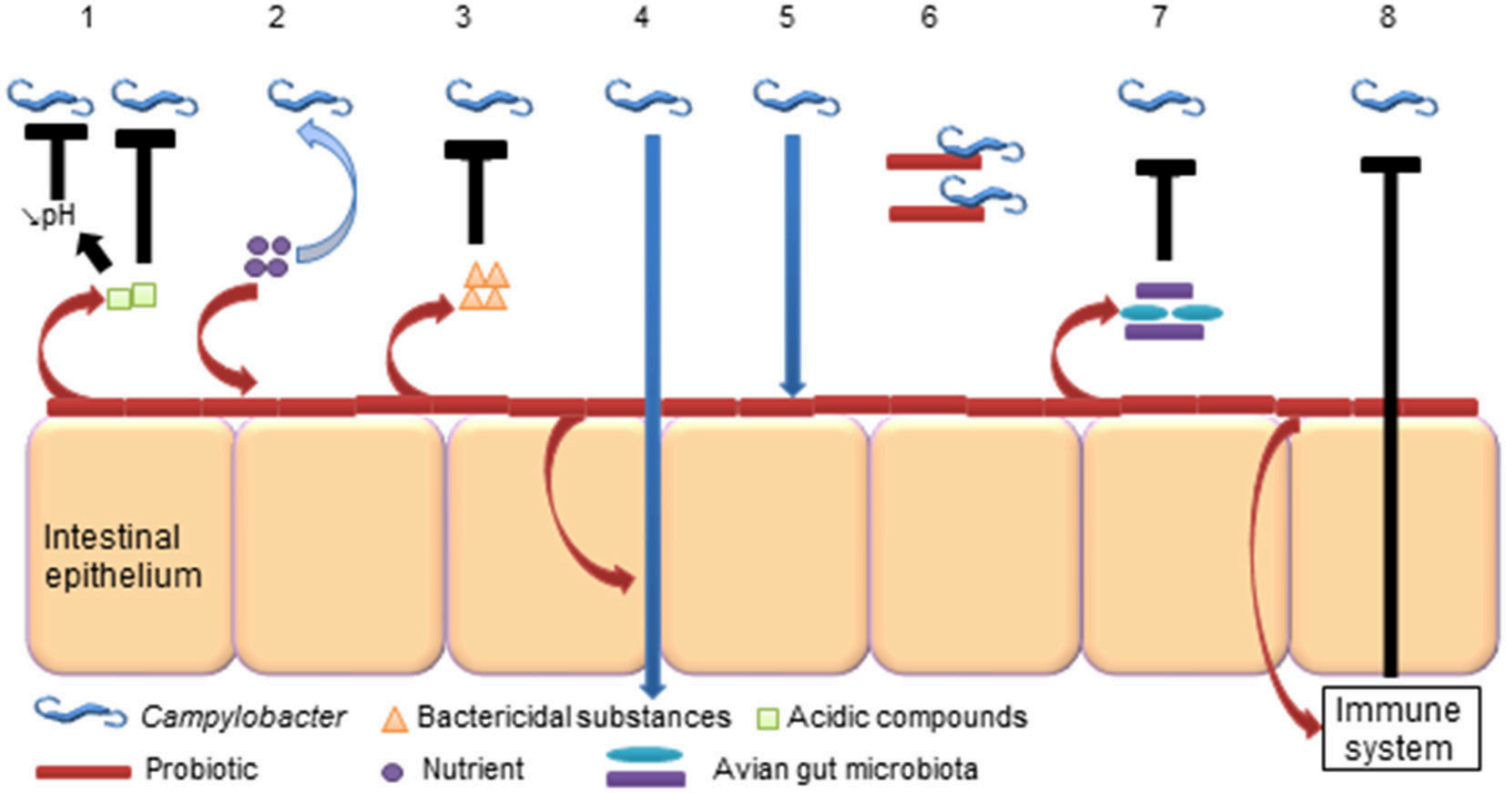
**Potential probiotic abilities to reduce *Campylobacter* in the avian gut**. (1) Probiotics produce acidic compounds (lactic acid), which could inhibit *Campylobacter* and reduce the gut luminal pH that could affect *Campylobacter* (Neal-McKinney et al., 2012). (2) Probiotics compete for nutrients with *Campylobacter* (Aho et al., 1992). (3) Probiotics produce bactericidal substances (bacteriocins, H_2_O_2_) that could kill *Campylobacter* (Messaoudi et al., 2012a). (4) Probiotics strengthen tight junctions of intestinal epithelium and prevent *Campylobacter* translocation (Messaoudi et al., 2012b). (5) Probiotics colonize intestinal epithelium and prevent adhesion and invasion of *Campylobacter* (Wine et al., 2009). (6) Probiotics bind *Campylobacter* (Nishiyama et al., 2014). (7) Probiotics alter the avian gut microbiota, which could affect *Campylobacter* colonization (Sanders, 2011). (8) Probiotics modulate the immune system, which acts against *Campylobacter* (Brisbin et al., 2011).

As probiotics inhibit foodborne pathogens such as *Salmonella* (Nurmi and Rantala, 1973), often designated as competitive exclusion, they could potentially have an effect on *Campylobacter* (Figure 2). Indeed, probiotic bacteria successfully excluded *C. jejuni* from mice (Sorokulova et al., 1997; Wagner et al., 2009). Regarding chickens, potential probiotic mechanisms associated with the inhibition of *Campylobacter* have been reviewed and detailed by Mohan (2015).

**Figure 3:**
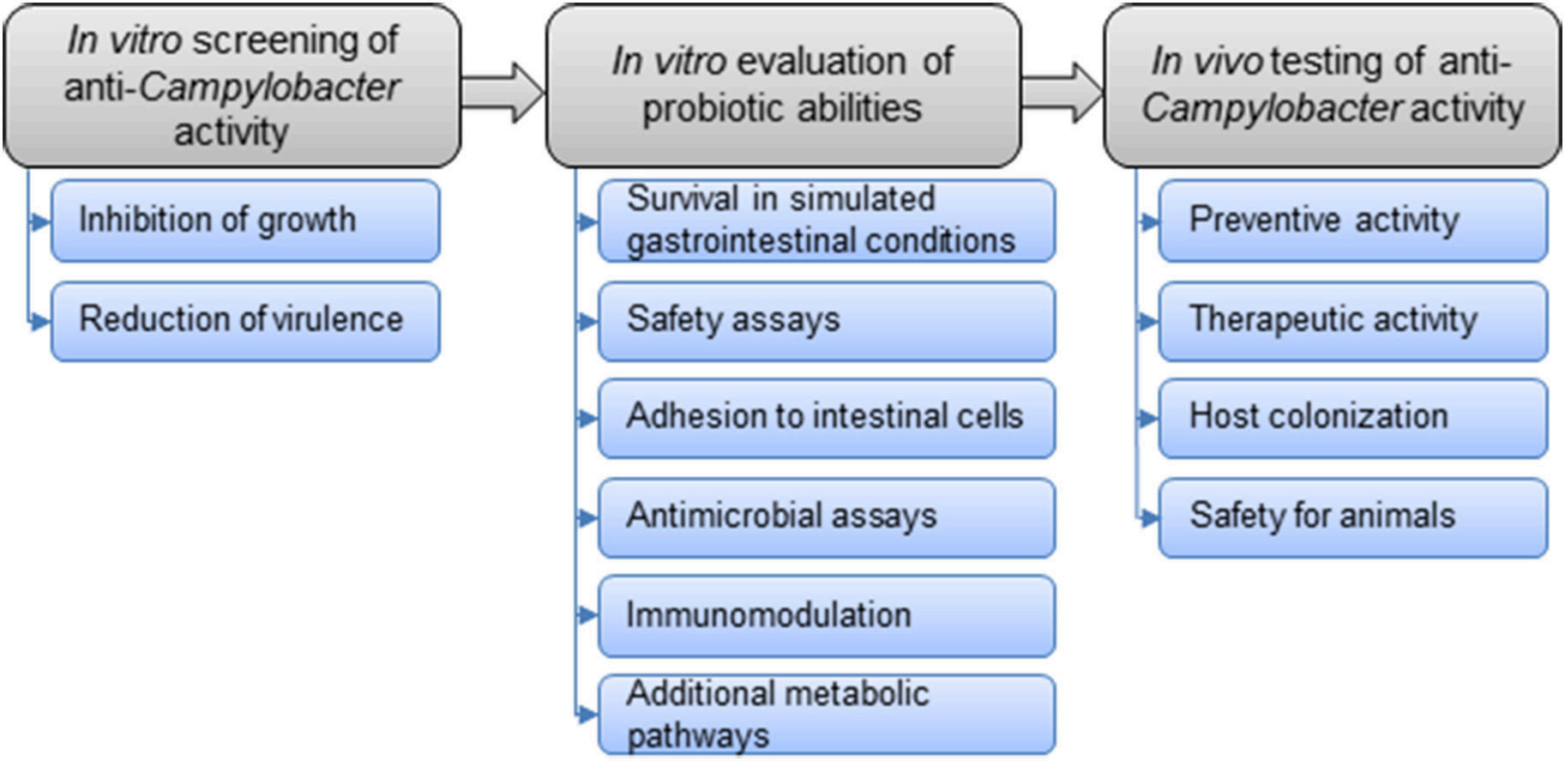
**Overall strategy to select potential probiotics to control *Campylobacter* in chickens**.

**Figure 4:**
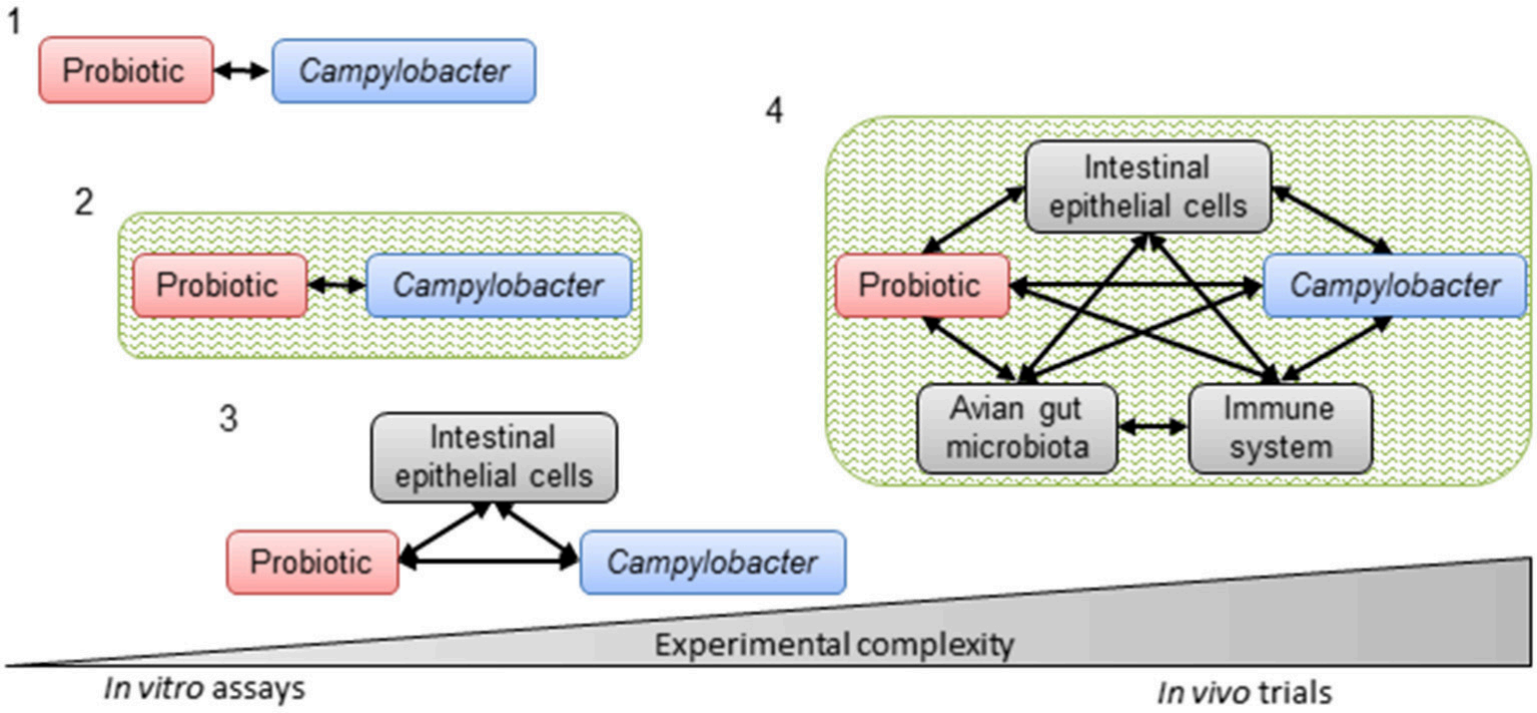
**Progressive complexity of methods used to select probiotics with *in vitro* and *in vivo* anti-*Campylobacter* activity**. Simplified *in vitro* assays to assess direct interactions between the probiotic and *Campylobacter* (1) without (co-culture and agar plate diffusion) or (2) with an intestinal environment (co-culture in batch) or (3) interactions between the probiotic, *Campylobacter* and intestinal epithelial cells (adhesion and invasion assays). Complex *in vivo* trials (4) with their potential interactions to corroborate *in vitro* assays. Black arrows represent potential interactions. Green represents the intestinal environment.

The general strategy for the selection of probiotic strains requires a set of experiments to identify the most promising candidates (Figure 3). *In vitro* studies include aggregation, co-aggregation, cell surface hydrophobicity and adhesion activities on epithelial cells. Additionally, growth with bile acids (chicken bile) and tolerance to acidic pH are checked. In addition to *in vitro* assays, *in vivo* experiments on chickens are carried out to highlight the impact of probiotics on foodborne pathogen colonization and/or the effect on growth performances in animals. This strategy includes simplified *in vitro* assays for probiotic screening, followed by more complex *in vivo* trials to confirm the anti-*Campylobacter* activity (Figure 4).

## *In vitro* screening for anti-*Campylobacter* probiotics

*In vitro* studies are preliminary screening tools for the selection of potential probiotic cultures, and the first stage for further application in poultry production. Based on tests that confirm some antimicrobial properties, several potential anti-*Campylobacter* bacteria have been isolated (Table 2). A whole set of experiments can be carried out to identify the mechanism involved in the anti-*Campylobacter* activity. The ability to inhibit the pathogen's growth can be evaluated by co-culture experiments as well as by antimicrobial assays with cell-free culture supernatant, while interference with the adhesion to/invasion of intestinal cells can be studied by adhesion and invasion inhibition assays.

**Table 2:** ***In vitro* experiments related to the probiotic impact on *Campylobacter***.

**Study**	**Probiotic agents**	***C. jejuni* strain (origin)**	**Test**	**Epithelial cells or mucus**	**Temperature**	**Time of incubation**	**Observed effects (results)**	**Mechanism involved**
**PATHOGEN + PROBIOTIC**								
Fooks and Gibson, 2003	*Lb. plantarum 0407* + oligofructose *Bf. bifidum* Bb12 + oligofructose + xylo-oligosaccharides	CIP 70.2 (bovine)	Co-culture in batch and continuous culture anaerobic fermentation systems	–	37°C	24 h	Growth inhibition (8 log reduction)	Lactic and acetic acid production
Fernández et al., 2003	*Lb. acidophilus* UO 001 *Lb. gasseri* UO 002	Clinical isolate (human)	Agar plate diffusion	–	37°C	48 h	Inhibition zone (NI)	Lactic acid production
Chaveerach et al., 2004	*Lactobacillus* spp. P93	C2146 (chicken) C186 (chicken) C350 (chicken) C591 (chicken) C690 (chicken) C144 (chicken)	Co-culture and agar plate diffusion	–	37°C	72 h	Growth inhibition (4–6 log reduction) Inhibition zone (9–15 mm)	Organic acid and bacteriocin production
Messaoudi et al., 2011	*Lb. salivarius* SMXD51 *Lb. salivarius* MMS122 *Lb. salivarius* MMS151	NCTC 11168 (human) 81–176 (human)	Agar plate diffusion	–	37°C	24 h	Inhibition zone (NI)	Bacteriocin production
Dubois Dauphin et al., 2011	*E. faecium* THT *Lb. pentosus* CWBI B78	LMG 6446 (human) CWBI B1444 (NI)	Co-culture and agar plate diffusion	–	37°C	100 h	Inhibition zone (10–15 mm)	Lactic and acetic acid production
Robyn et al., 2012	*E. faecalis* MB 5259	MB 4185 (chicken)	Co-culture in batch and agar plate diffusion	–	37°C	48 h	Growth inhibition (0.5–1 log reduction) Inhibition zone (NI)	NI
Mundi et al., 2013	*Lb. acidophilus* La-5 *Bf. longum* NCC2705	81–176 (human)	*Campylobacter* culture with neutralized cell-free supernatants from probiotics	–	42°C	2 h	Virulence gene down-regulation (3–7-fold reduction for *ciaB* and *flaA* genes)	Biologically active molecules production
Menconi et al., 2014	*Pediococcus parvulus* *Lb. salivarius*	NI	Agar plate diffusion	–	37°C	24 h	Inhibition zone (NI)	NI
Bratz et al., 2015	*Lb. fermentum* ATCC 14931 *Lb. johnsonii* BFE 663 *Lb. paracasei* IMT 22353	NCTC 11168 (human) CIP 70.2 (bovine)	Agar plate diffusion	–	37°C	24 h	Inhibition zone (NI)	Organic acid production
**PATHOGEN + PROBIOTIC + MUCUS**								
Ganan et al., 2013	*Propionibacterium freudenreichii* DSM 7067 *Lb. rhamnosus* ATCC 53103 *Lactococcus lactis* N8 Broilact® (facultative anaerobic bacteria)	NCTC 11168 (human) 118 (human)	Adhesion assay (exclusion test^a^, competition test^b^)	Chicken intestinal mucus	37°C	1 h	Adhesion reduction (8–23%)	Competition for adhesion site
Tareb et al., 2013	*Lb. rhamnosus* CNCM-I-3698 *Lb. farciminis* CNCM-I-3699	CIP 70.2 (bovine)	Adhesion assay (exclusion test^a^, competition test^b^)	Mucin	37°C	1 h	Adhesion reduction (17–70%)	Co-aggregation
**PATHOGEN + PROBIOTIC + EPITHELIAL CELLS**								
Wine et al., 2009	*Lb. helveticus* R0052 *Lb. rhamnosus* R0011 *Lb. rhamnosus* ATCC 53103	NCTC 11168 (human) 81–176 (human)	Invasion assay (exclusion test^a^, competition test^b^)	T84 INT-407	37°C	1 or 4 h (probiotics) 4 h (*Campylobacter*)	Invasion reduction (35–55%)	Competition for adhesion site
Alemka et al., 2010	*Lb. rhamnosus* R0011 *Lb. helveticus* R0052 *Lb. salivarius* AH102 *Bf. longum* AH1205 Lacidofil® *(Lb. rhamnosus* R0011 + *Lb. helveticus* R0052) Mixture (*Lb. rhamnosus + Lb. helveticus + Lb. salivarius*)	81–176 (human)	Invasion assay (exclusion test^a^)	HT29-MTXE12 HT29	37°C	4 or 15 h (probiotics) 24 h (*Campylobacter*)	Invasion reduction (1–1.5 log) and translocation reduction (3–4 log)	NI
Campana et al., 2012	*Lb. acidophilus* ATCC 4356	Hom 107 (human) ISS 9 (human) ISS 3 (human) Hom 13 (human) 241 (human) ISS 1 (human) Hom 88 (human) Hom 14 (human) Hom 7 (human)	Adhesion and invasion assays (exclusion test^a^, competition test^b^, displacement test^c^)	Caco-2	37°C	1 or 4 h (probiotics) 4 h (*Campylobacter*)	Adhesion reduction (10–50%) and invasion reduction (10–50%)	Competition for adhesion site Bacteriocin production
Wang et al., 2014	*Lb. plantarum* N8 *Lb. plantarum* N9 *Lb. plantarum* ZL5 *Lb. casei* ZL4	NCTC 11168 (human) ATCC 33291 (human) ATCC BAA-1153 (human)	Adhesion and invasion assays (exclusion test^a^, competition test^b^, displacement test^*c*^)	HT29	37°C	1 or 4 h (probiotics) 4 h (*Campylobacter*)	Adhesion reduction (40–70%) and invasion reduction (30–60%)	Organic acid and bacteriocin production

*Lb, Lactobacillus; Bf, Bifidobacterium; E, Enterococcus; NI, not indicated*.

a *Probiotics were incubated before Campylobacter to assess a preventive effect*.

b *Probiotics and Campylobacter were incubated at the same time to assess a therapeutic effect*.

c *Campylobacter were incubated before probiotics to assess a therapeutic effect*.

*Lacidofil® is produced by Xymogen (Orlando, FL, USA); Broilact® is produced by Nimrod Veterinary Products, (Gloucester, UK)*.

### Probiotic identification

Identification of probiotic strain at species level is still important as the GRAS (Generally Recognized As Safe) and QPS (Qualified Presumption of Safety) status defined in USA and Europe, respectively, are both based on the species name. Traditional methods for bacterial identification and phenotypical characterization, such as API system, BIOLOG or culture-based techniques can be used to identify probiotics strains (Herbel et al., 2013; Bagheripoor-Fallah et al., 2015; Galanis et al., 2015; Cherdyntseva et al., 2016). For instance, the main phenotypic methods for *Lactobacillus* probiotic identification were discussed in Herbel et al. (2013). However, these conventional microbiological tests may have limitations in discriminating large numbers of isolates with similar physiological characteristics (Herbel et al., 2013; Bagheripoor-Fallah et al., 2015; Yadav and Shukla, 2015). In addition, culture-based techniques provide strains able to replicate under experimental conditions, indeed selective media exist only for a limited subset of potential strains of interest (Davis, 2014).

Several DNA-based techniques have been developed to overcome this obstacle (Bagheripoor-Fallah et al., 2015; Yadav and Shukla, 2015), such as the pulsed field gel electrophoresis (PFGE) mainly used for probiotic strain differentiation and discrimination (Tynkkynen et al., 1999; Gosiewski and Brzychczy-Wloch, 2015). However, it cannot be applied for direct detection of a particular strain, in a single reaction (Tynkkynen et al., 1999). Moreover, it is laborious, time-consuming and, thus, inappropriate for large scale screening experiments from environmental samples, especially when microbial groups, other than those needed to be identified, are at higher population levels. In addition, the PCR methodology (mostly on 16S and 23S ribosomal RNA) coupled to sequencing is commonly employed for efficient identification of lactic acid bacteria (Allegretti et al., 2014; Yadav and Shukla, 2015; Cherdyntseva et al., 2016). It is easy to implement, fast, cost efficient, and requires a small amount of template DNA. However, when the design of specific primers is not feasible, the random amplified polymorphic DNA (RAPD) technique may be applied. RAPD is a PCR-based assay that uses short arbitrary primers that anneal to multiple random target sequences to generate the needed polymorphism (Galanis et al., 2015). A recent article published by Yadav and Shukla (2015) reviewed molecular and analytical techniques to identify and screen probiotics. Among the methods discussed, quantitative analysis by real-time PCR (RT-PCR or qPCR) and fluorescent based-methods (fluorescent *in situ* hybridization and fluorescent activated cell sorting) enables the discrimination of different species and to quantify the amount of bacteria used in a sample (Herbel et al., 2013; Yadav and Shukla, 2015). These last years, development of new techniques to improve bacterial strain identification and characterization is facilitated by the next-generation sequencing (NGS) technologies (Herbel et al., 2013). These techniques would allow identification of non-cultivable strains and also analyze of metabolites produced by probiotics by metabolomics. In addition, whole genome sequencing (WGS) offers an insight regarding evolutionary background and diversity of lactic acid bacteria belonging to one species (Herbel et al., 2013). For example, comparative genome analysis of published *Lb. salivarius* sequences led to the identification several genes known to be important for gastrointestinal survival, adherence to cells, and bacteriocin production in *Lb. salivarius* SMXD51 (Kergourlay et al., 2012).

### Growth inhibition assays

This first step of screening consists of monitoring *Campylobacter* growth in the presence of the probiotic in a co-culture assay or its supernatant in an agar plate diffusion assay (Table 2). This method is easy, applicable to a large number of test strains and, in addition, does not require expensive laboratory equipment. Using this approach, *Lb. acidophilus* and *Lb. gasseri* have been shown to inhibit strongly *C. jejuni* by lactic acid production (Fernández et al., 2003). Similarly, the ability of *Lactobacillus* spp. isolated from chickens to inhibit the growth of *C. jejuni* has been demonstrated by Chaveerach et al. (2004). These results suggest that the inhibitory effect of *Lactobacillus* strains on *Campylobacter* growth is a combination of organic acid and bacteriocin production (Chaveerach et al., 2004). These findings have been supported by Dubois Dauphin et al. (2011) who observed the antimicrobial effect of *E. faecium* THT due to lactic acid production and *Lb. pentosus* CWBI B78 due to lactic and acetic acid production. Messaoudi et al. (2011) identified three *Lb. salivarius strains*, i.e., SMXD51, MMS122, and MMS151, from chicken ceca with antagonism against *C. jejuni* strains NCTC 11168 and 81–176 due to the production of bacteriocins. Recently, *Lb*. *fermentum* ATCC 1493, *Lb*. *johnsonii* BFE 663 and *Lb*. *paracasei* IMT 22353 showed antimicrobial activity against *C*. *jejuni* NCTC 11168 and *C*. *jejuni* CIP 70.2 (Bratz et al., 2015). It turned out that the anti-*Campylobacter* activity of the *Lactobacillus* strains was pH-dependent, i.e., pH < 4.3.

*In vitro* fermentation experiments under controlled temperature, pH and atmosphere were carried out to elucidate further the ability of probiotics to inhibit *Campylobacter* growth under conditions simulating those in broiler ceca. Chang and Chen (2000) demonstrated an antagonistic effect on *C. jejuni* by four lactobacilli, including *Lb*. *acidophilus, Lb*. *fermentum, Lb*. *crispatus*, and *Lb*. *brevis*, in a complete simulated digestive tract model. Similarly, Robyn et al. (2012) showed the *in vitro* anti-*Campylobacter* activity of *E*. *faecalis* MB 5259. Even though the model mimics the broiler cecal environment, i.e., pH and bile salts, and anaerobic incubation, a major limitation of this approach is the lack of epithelial cells and avian gut microbiota that compose the intestine.

### Adhesion and invasion inhibition assays

The inhibition assays described in Section Growth Inhibition Assays are not solely suitable to confirm the anti-*Campylobacter* effect of probiotics because these experiments do not take into account the complexity of interactions occurred *in vivo*, whose interaction with the epithelial intestinal cells. Thus, a better characterization of the mechanisms of action of probiotic strains on *Campylobacter* is required. Another screening step is to test the ability of the probiotic strain to inhibit or modulate *Campylobacter* infection in epithelial intestinal cells. Probiotic and pathogen are incubated with intestinal monolayer cells and then all the pathogens that adhere to and invade eukaryotic cells are enumerated in order to determine the adhesion and invasion indexes. In addition, the number of probiotic cells that adhered to the monolayer could be also counted to assess the adhesion ability of the probiotic strain. The possible impact of the probiotic on the structure and integrity of the eukaryotic cells could be also evaluated to provide useful information on the mode of action of the probiotic.

Although the purpose of the *in vitro* experiments presented in the Table 1 is to highlight the anti-*Campylobacter* activity of probiotics for further application at the farm level, particularly in poultry farms, no *in vitro* experiments including avian intestinal cell lines have been carried out as, to our knowledge, these cell lines are not yet commercialized. For example, Van Deun et al. (2008) used ceca from commercial brown laying hens at the age of 12–20 weeks to isolate primary epithelial cells from crypts according to a modified protocol of Booth et al. (1994), which requires specialized expertise (Booth et al., 1994; Van Deun et al., 2008). It is worth noting that the LMH cell line (Kawaguchi et al., 1987) is the only chicken epithelial cell line currently available to researchers from the ATCC culture collection (Larson et al., 2008). LMH is a primary hepatocellular carcinoma epithelial cell line and has been used previously as an *in vitro* model to investigate mechanisms of *C. jejuni* colonization in poultry (Smith et al., 2005; Byrne et al., 2007). Although the LMH chicken epithelial cells are derived from the liver, the results obtained with this cell line *in vitro* were correlated with *in vivo* findings (Konkel et al., 2007). In addition, Smith et al. (2005) and Byrne et al. (2007) reported that *C. jejuni* isolates invade chicken primary cells and human cells at comparable levels. In contrast to these results, Larson et al. (2008) found that *C. jejuni* invades chicken LMH epithelial cells in significantly lower numbers (0.6–1.7 log differences) than it invades human INT 407 epithelial cells, although the bacterial adhesion assays showed that *C. jejuni* adhere to LMH cells and INT 407 cells in comparable numbers. The chicken LMH cell line has also been used to evaluate the adhesion of *Lactobacillus* cultures to epithelial cells (Spivey et al., 2014). Thus, LMH epithelial cells may represent an alternative cell line for the investigation of probiotic functionality and mechanistic studies, but efforts should be made to develop a stable avian intestinal cell line.

On the contrary, the *in vitro* human cell lines are well established and have been used for many years to investigate specific aspects of small intestinal function. They could reflect the interaction between the pathogen and the probiotic bacteria. They are useful for the evaluation of the immunomodulation activity of probiotic strains, by assaying cytokine production (Ashraf and Shah, 2014; Vitaliti et al., 2014; Frei et al., 2015). Moreover, as shown previously, several steps of *C. jejuni* pathogeny, including adhesion, invasion and translocation, could be assessed using these cell lines (Haddad et al., 2010a,b). Thereby, this model may help to clarify whether probiotic strains prevent or reduce damage to epithelial integrity caused by a pathogenic challenge. Although this model does not completely reflect the *in vivo* setting, it does provide a valuable opportunity to study the interactions between the enteric pathogen, potentially beneficial microorganisms, and host epithelial cells.

The different experiments involving epithelial cells are described and summarized in Table 2. Most studies showed slight reductions in adhesion and invasion ranging from 8 to 70%. For example, a 55% reduction in the invasion of human intestinal epithelial cells by *C. jejuni* was observed after treatment with *Lb. helveticus* R0052, which suggested that competitive exclusion could contribute to protection by adherent probiotics (Wine et al., 2009). One important point highlighted by these authors is the strain specificity of the described effects. Their results demonstrated that *Lb. helveticus* R0052 is more effective than either *Lb. rhamnosus* R0011 or *Lb. rhamnosus* GG in interfering with *C. jejuni* invasion of intestinal epithelial cells. This observation highlights the complexity of the interactions between microorganisms and mammalian cells.

Similarly, probiotics attenuated *C. jejuni* association with and internalization within HT29-MTXE12 cells, and translocation of the bacteria to the basolateral medium of transwells (Alemka et al., 2010). The studies mentioned above emphasized probiotics as a preventive/protective measure to limit *Campylobacter* infection. Interestingly, HT29-MTXE12 cells are a cell line that provides an opportunity to study the role of mucus *in vitro*, and the relationship of mucus-associated factors with the anti-*Campylobacter* activity of probiotics, as *Campylobacter* inhabits the mucus layer in the avian host (Van Deun et al., 2008). Simplified models with mucin (Tareb et al., 2013) or chicken intestinal mucus (Ganan et al., 2013) showed that probiotics were able to reduce the binding of *Campylobacter* spp. when the probiotics colonized the mucus before the pathogen (Table 2).

Campana et al. (2012) also observed the inhibitory properties of *Lb. acidophilus* ATCC 4356 on Caco-2 cell adhesion to/invasion of *C. jejuni*. More recently, Wang et al. (2014) isolated four adhesive *Lactobacillus* strains able to exert significant antagonistic activity against *C. jejuni in vitro* and to promote effective inhibition of the adhesion to and invasion of HT29 cells by *C. jejuni*. Their bactericidal capacity is probably related to the low pH and the production of metabolites, such as lactic acid and antibiotic-like substances. These last two works emphasized the beneficial effects of probiotics not only as a preventive/protective measure but also as a therapeutic one.

Several limitations of these kinds of experiment need to be reported. *C. jejuni* isolates from humans, chickens or pigs are capable of adhering to and invading human, avian and porcine cell lines (Biswas et al., 2000; Gripp et al., 2011) with different efficiencies (Poly et al., 2007; Larson et al., 2008; Wine et al., 2008). Moreover, the capabilities between strains vary significantly (Newell et al., 1985; Fauchere et al., 1986; Biswas et al., 2000; Fearnley et al., 2008; Zheng et al., 2008). It is also well-known that *Campylobacter* spp. exhibit high genetic and phenotypic variability and flexibility (Gripp et al., 2011; Rodrigues et al., 2015; Bronnec et al., 2016), and, as a consequence, are not equally virulent and probably not equally sensitive to probiotic actions (Wine et al., 2009).

Another limitation is the growth conditions, which are very beneficial for the bacteria but do not reflect a realistic intestinal environment. Similarly, in almost all the studies described in Table 2, experiments and strain cultures were carried out at 37°C, which is not in accordance with the temperature of chicken, i.e., 42°C. This difference could have an impact on the *in vitro* anti-*Campylobacter* activity. For example, it has been documented that bacteriocin production can be sensitive to environmental changes and parameters including temperature, pH and growth medium (Cintas et al., 2000; Diep et al., 2000; Qi et al., 2001). Therefore, the models used are not optimized to characterize completely *C. jejuni* virulence or colonization, and thus the effect of probiotics on the infection biology of this pathogen.

Despite these limitations, the techniques mentioned (Table 2) are relevant for initially screening probiotic strains with anti-*Campylobacter* activity and speculating on the mechanisms involved. However, improved adhesion and invasion inhibition assays would use an avian intestinal cell line that secretes mucus, an incubation temperature of 42°C (temperature of chicken), field *Campylobacter* strains (isolated from chicken), and a reference strain. This strain of choice should exhibit efficient adherence and invasion characteristics but also robustly infect animal models (Ahmed et al., 2002; Seal et al., 2007; Hiett et al., 2008). These strains, such as NCTC 11168, 81–176, RM1221 and 81116, are the most commonly used isolates in laboratories and have been successfully used for *in vitro* and *in vivo* infection studies. Such reference strains will ensure a critical comparison of the impact of probiotics between the different studies performed. In addition, complete genome sequences of these reference strains are available and could allow further investigations of the interactions between pathogens and probiotics at the genomic level (Parkhill et al., 2000; Fouts et al., 2005; Gundogdu et al., 2007; Pearson et al., 2007).

Nevertheless, the results obtained need to be confirmed by *in vivo* experiments because *in vitro* experiments do not take into account major parameters (Figure 4), such as avian gut microbiota, immune response and feed, which could interact with the probiotic and its anti-*Campylobacter* activity.

## Anti-*Campylobacter* activity of probiotics in broilers

Many early reports showed that the administration of probiotics, especially *Lactobacillus* and *Bifidobacterium*, improved growth performances in animals such as broilers by increasing the utilization of nutrients (Jin et al., 1998). An overview of the effects of probiotics was given by Oelschlaeger (2010). Among them, the exclusion of pathogens (Tsai et al., 2005) seems to be a valid approach to counteract foodborne pathogen contamination. Published *in vivo* studies, summarized in Table 3, have pointed out a possible role of probiotics in preventing the shedding of *C. jejuni* at the level of primary production.

**Table 3:** ***In vivo* studies using probiotics to reduce *Campylobacter* colonization in broilers**.

**Study**	**Probiotic administration**				***C. jejuni*** **contamination**					***C. jejuni*** **enumeration**	
	**Strain**	**Dose**	**Route**	**Period**	**Type**	**Chicken age or period**	**Strain (origin)**	**Dose**	**Organ**	**Chicken age**	Results^*^
**PROBIOTIC ALONE**											
Netherwood et al., 1999	*E. faecium* NCIMB 11508 transformed with plasmid pVACMC1 containing the *Ruminococcus flavefaciens* b-1,4-glucanase gene	1.10^6^ CFU/chick	*Per os*	From day of hatching to day 28	Natural	–	–	–	Crops, duodena, ceca	14, 28, 30, 33, and 35 days	No reduction
Fritts et al., 2000	Calsporin® *(B. subtilis* C-3102)	NI	Diet	From day of hatching to day 42	Natural	–	–	–	Processed carcasses	42 days	0.2 log reduction
Line et al., 2008	*Lb. salivarius* NRRL B-30514 *Paenibacillus polymyxa* NRRL B-30509	2.10^8^ CFU/chick	*Per os*	From day of hatching to day 4 twice daily	Artificial	From day of hatching to day 10	NI	1.10^3^, 1.10^4^ or 1.10^5^ CFU/chick	Ceca	7 days	No reduction
Santini et al., 2010	*Bf. longum* PCB133	1.10^8^ CFU/chick	*Per os*	From day of hatching to day 14	Natural	–	–	–	Feces	15 days	1 log reduction
Neal-McKinney et al., 2012	*Lb. crispatus* JCM5810	1.10^8^ CFU/chick	*Per os*	4 days	Artificial	14 days	F38011 (human)	1.10^8^ CFU/chick	Ceca	21 days	2 log reduction
Robyn et al., 2013	*E. faecalis* MB5259	1.10^4^ or 1.10^8^ CFU/chick	*Per os*	NI	Artificial	15 days	MB 4185 (chicken)	2.10^4^ CFU/chick	Ceca	21 days	No reduction
Nishiyama et al., 2014	*Lb. gasseri* SBT2055	1.10^8^ CFU/chick	*Per os*	From day of hatching to day 15	Artificial	1 day	81–176 (human)	1.10^6^ CFU/chick	Ceca	15 days	2 log reduction
Arsi et al., 2015a	*Bacillus* spp.	NI	Intracloacal or *per os*	Day of hatching	Artificial	7 days	Four-strain mixture	1.10^6^ CFU/chick	Ceca	14 days	1–3 log reduction (intracloacal) No reduction (*per os*)
Arsi et al., 2015b	*Bacillus* spp. *Lb. salivarius subsp. salivarius Lb. salivarius subsp. salicinius*	2.10^6^ CFU/chick	*Per os*	Day of hatching	Artificial	7 days	Four-strain mixture	1.10^6^ CFU/chick	Ceca	14 days	1–3 log reduction
Nishiyama et al., 2015	*Lb. gasseri* SBT2055	1.10^8^ CFU/chick	*Per os*	From day of hatching to day 15	Artificial	1 day	81–176 (human)	1.10^6^ CFU/chick	Ceca	15 days	1–2 log reduction
Gracia et al., 2016	*B. subtilis* DSM17299	0.05% (w/w)	Diet	From day of hatching to day 42	Artificial	14 days	Isolate from ST45 complex (chicken)	1.10^4^ CFU/chick	Ceca	21, 35 and 42 days	No reduction
Guyard-Nicodème et al., 2016	Calsporin® Ecobiol® (*B. amyloliquefaciens*)	0.01% (w/w) 0.1% (w/w)	Diet	From day of hatching to day 42	Artificial	11 days	C97ANSES640 (chicken)	1.10^4^ CFU/chick	Ceca	14, 35 and 42 days	1.7 log reduction at 42 days (Calsporin®) No reduction (Ecobiol®)
**PROBIOTIC MIXTURE**											
Aho et al., 1992	K-bacteria (microaerophilic adaptive-mucus bacteria) + Broilact® (facultative anaerobic bacteria)	NI	Water	From day of hatching to day 38	Artificial	4 days	T23/42 (chicken)	1.10^4^ CFU/chick	Ceca	38 days	1.5–2 log reduction
Schoeni and Wong, 1994	*Citrobacter diversus* 22 + *Klebsiella pneumonia* 23 + *Escherichia coli* 25 + mannose	1.10^8^ CFU/chick	*Per os*	Days 1 and 3	Artificial	1 day	108 (chicken)	1.10^8^ CFU/chick	Ceca	7 days	62% reduction in the colonization rate
Morishita et al., 1997	Avian PAC Soluble®; *(Lb. acidophilus* + *Streptococcus faecium)*	400 mg/L	Water	From day of hatching to day 3	Artificial	1 day	C101 (chicken)	1.10^4^ CFU/chick	Cloacal swabs	39 days	70% reduction in prevalence
Willis and Reid, 2008	Starter diet (*Lb. acidophilus + Lb. casei + Bf. thermophilus + E. faecium*)	1.10^8^ CFU/kg of feed	Diet	From day of hatching to day 42	Natural	–	–	–	Cloacal swabs	42 days	10% reduction in prevalence
Baffoni et al., 2012	Microencapsulated *Bf. longum* PCB133 + oligosaccharides	1.10^9^ CFU/chick + 3% of galactooligosaccharide (w/w)	Diet	From day of hatching to day 14	Natural	–	–	–	Feces	15 days	0.5 log reduction
Ghareeb et al., 2012	PoultryStar sol® (*E. faecium + P. acidilactici + Bf. animalis + Lb. salivarius + Lb. reuteri*)	2 or 20 mg	Water	From day of hatching to day 14	Artificial	1 day	3015/2010 (chicken)	1.10^4^ CFU/chick	Ceca	15 days	3.7–5.5 log reduction
Aguiar et al., 2013	Three *B. subtilis* sp. mixture	2.10^6^ CFU/chick	*Per os*	Day of hatching	Artificial	7 days	Four-strain mixture (chicken)	1.10^5^ CFU/chick	Ceca	14 days	1–4 log reduction
Cean et al., 2015	*Lb*. *paracasei* J.R + *Lb*. *rhamnosus* 15b + *Lb*. *lactis* Y + *Lb*. *lactis* FOa	NI	Water	From day of hatching to day 42	Natural	–	–	–	Duodena, ceca, feces	42 days	5 log reduction (duodena and ceca)
Guyard-Nicodème et al., 2016	PoultryStar ME®	0.1% (w/w)	Diet	From day of hatching to day 42	Artificial	11 days	C97ANSES640 (chicken)	1.10^4^ CFU/chick	Ceca	14, 35 and 42 days	0.5 log reduction at 14 days and 1.9 log reduction at 35 days

* *Only results with statistical reduction are presented*.

*E, Enterococcus; B, Bacillus; Lb, Lactobacillus; Bf, Bifidobacterium; P, Pediococcus; NI, not indicated; Calsporin® is produced by Calpis (Tokyo, Japan); Broilact® is produced by Nimrod Veterinary Products (Gloucester, UK); PoultryStar sol® and PoultryStar ME® are produced by BIOMIN (Holding GmbH, Getzersdorf, Austria); Avian PAC Soluble® is produced by Pacific Agri-Sales (Visalia, CA, USA); Ecobiol® is produced by Norel Animal Nutrition (Madrid, Spain)*.

### Probiotics used alone

Several studies suggest that a therapeutic treatment could be useful in suppressing *C. jejuni* colonization of chicks at early growth stages (Table 3). Birds fed diets including *Bacillus subtilis* C-3102 had significantly reduced numbers of *Campylobacter* (0.2 log) than birds fed with the control diet (Fritts et al., 2000). Neal-McKinney et al. (2012) found that the number of *C. jejuni* was reduced by almost two orders of magnitude in commercial broiler chickens fed with *Lb. crispatus* showing a potential role of the probiotic as a preventive/protective measure. After investigating possible mechanisms for this reduction, including production of bacteriocins, stimulation of antibody production, alteration of the cecal microbiome, and production of lactic acid, the authors concluded that only the production of lactic acid was supported by their data (Neal-McKinney et al., 2012). Recently, Nishiyama et al. (2014) demonstrated the ability of *Lb. gasseri* SBT2055 to inhibit the adhesion and invasion of *C. jejuni in vitro* and *C. jejuni* colonization of chicks *in vivo*. Their data suggested a pivotal role for APF1 in mediating the interaction of LG2055 with human intestinal cells and in inhibiting *C. jejuni* colonization of the gastrointestinal tract (Nishiyama et al., 2015). Recently, Arsi et al. (2015a) collected bacterial isolates (*Bacillus* spp.) with anti-*Campylobacter* activity *in vitro* and evaluated their efficacy *in vivo* after oral or intracloacal inoculation into chicks. They demonstrated that, when dosed orally, only one isolate had a 1 log reduction in cecal *Campylobacter* counts, whereas when administered intracloacally, six isolates produced a 1–3 log reduction in cecal *Campylobacter* counts in 14-day-old chickens (Arsi et al., 2015a). Their results highlight the fact that if probiotics are protected during transit through the upper gastrointestinal tract and are thus available in the lower intestinal tract, they could reduce *Campylobacter* colonization in broiler chickens. This is also the first study to show an anti-*Campylobacter* effect of a single probiotic on a four-strain mixture of *Campylobacter*.

Contrary to these results, treatments with viable probiotic bacterial cultures (*Lb. salivarius* NRRL B-30514 or *Paenibacillus polymyxa* NRRL B-30509) were ineffective in reducing *C. jejuni* in chickens, using both prophylactic or therapeutic administration (Line et al., 2008), while treatment with bacteriocins from these corresponding bacteria substantially reduced *C. jejuni* colonization in live chickens (Svetoch et al., 2005). Finally, this anti-*Campylobacter* activity of *P. polymyxa* was not due to a bacteriocin and was reassigned to the lipopeptide tridecaptin A_1_ (Lohans et al., 2014). Another study showed that *in vitro* activity of *Bf. longum* PCB 133 against *C. jejuni* was confirmed in *in vivo* trials while *Lb. plantarum* PCS 20 failed to show any efficacy (Santini et al., 2010). Netherwood et al. (1999) also showed no evidence of a beneficial effect on the shedding of *Campylobacter* by chickens treated with the probiotic *E. faecium* NCIMB 11508. This result was corroborated by Robyn et al. (2013) with another *Enterococcus* strain. No evidence for inhibition was identified after challenging the probiotic *E. faecalis* MB 5259 with *Campylobacter* in broilers, although an *in vitro* inhibitory influence of the *E. faecalis* strain on *C. jejuni* had previously been shown in a system mimicking the broiler cecal environment (Robyn et al., 2013).

As a general remark, studies using probiotics individually have demonstrated heterogeneous results (Table 3). The presence of the complex avian gut microbiota, which could interact with the anti-*Campylobacter* activity, might partly explain this difference between *in vitro* and *in vivo* results. Thus, an effective alternative can be combinations of probiotic strains, which individually show anti-*Campylobacter* activities such as aggregation, competition for site adhesion, bacteriocin and acid production.

### Probiotic mixture

In their search for a competitive flora against *Campylobacter*, Aho et al. (1992) isolated two K-bacteria, i.e., two strains of *Campylobacter*-like organisms, from the ceca of an adult hen. They found that these mucin-adapted microaerophilic bacteria combined with Broilact® (Nimrod Veterinary Products, Gloucester, United Kingdom) a commercial mix of facultative anaerobic bacteria, delayed the onset of *Campylobacter* colonization by 1.5 weeks, and maintained a low level of colonization of 1.5–2 log_10_ CFU/g in broiler chickens (Aho et al., 1992). These bacteria, as a preventive/protective measure, may compete with *Campylobacter* for the same ecological niche in the intestinal ecosystem. Nevertheless, the problem with using undefined bacterial mixtures was that the antagonistic activities of the supplied bacteria were not well understood and presented the potential risk of introducing avian or human pathogens into the food chain (Stavric, 1992). This study appears to be in line with Schoeni and Wong (1994) who found that a defined mixture of *Citrobacter diversus, Klebsiella pneumoniae*, and *Escherichia coli* reduced the colonization of *Campylobacter* by 62% in chicken. Morishita et al. (1997) orally administered a mixture of *Lb. acidophilus* and *Streptococcus faecium* (isolated from chicken gut) to chickens in their drinking water for the first 3 days of life. Six hours after the first treatment, they orally challenged them with *C. jejuni*. Chickens receiving the treatment were significantly less colonized with *C. jejuni* (70% reduction) than those in the control group. Similarly, Willis and Reid (2008) showed a lower level of *C. jejuni* in broiler chickens fed with a standard diet supplemented with a probiotic mixture containing *Lb. acidophilus, Lb. casei, Bf. thermophilus*, and *E. faecium* (10^8^ CFU/g).

Ghareeb et al. (2012) infected 1-day-old broiler chicks, which then received 2 or 20 mg/chick per day of a commercialized probiotic *via* their drinking water for 15 days. The protective administration of the multispecies probiotic product, containing avian-derived *Enterococcus, Pediococcus, Lactobacillus*, and *Bifidobacterium* microorganisms, to broiler chickens reduced the cecal colonization by 3.8–5.5 log of *C. jejuni* at both 8 and 15 days post-challenge and may have changed their gut microbiota in a way that is beneficial to the health of consumers by reducing the number of *Campylobacter* (Ghareeb et al., 2012). Cean et al. (2015) went a step further and investigated the presence of the pathogen in the feces, duodenal and cecal content and the duodenal and cecal mucosa after a 42-day treatment with a combination of *Lb. paracasei* J.R., *Lb*. *rhamnosus* 15b, *Lb*. *lactis* Y, and *Lb*. *lactis* FOa. A significant reduction in the pathogen load from 0.5 to 5 log in both intestinal content and mucus colonization was observed (Cean et al., 2015). The highest effect of the mixture was observed in the duodenal content while the reduction in *Campylobacter* loads in the cecal content was the lowest. This observation highlights that probiotic activity may depend on the part of gastrointestinal tract considered and suggests that probiotic concentrations may be lower in the ceca than in the duodenum and/or *Campylobacter* may be better protected in the ceca. In addition, these probiotics were effective even when introduced in broiler feed 7 days before slaughter, thus as a therapeutic measure.

Recently, Baffoni et al. (2012) evaluated the therapeutic ability of a synbiotic mixture of *Bf. longum* PCB133 and prebiotic oligosaccharides to reduce the presence of *C. jejuni* in broiler chicken gut. In their *in vivo* experiment, *C. jejuni* quantification showed a 0.5 log decrease while total bifidobacteria were significantly increased after 2 weeks of treatment compared with the control group. On one hand, they speculated that the increased number of bifidobacteria, determined by prebiotic oligosaccharide intake, helps modulate the expression of *Campylobacter* genes involved in adhesion, as reported by Ding et al. (2005). On the other hand, the probiotic strain PCB133 exerts an anti-*Campylobacter* effect through antibacterial metabolite production, mainly acidic products, as well evidenced in the literature for other probiotic strains (Marianelli et al., 2010). These results illustrate the concept of synbiotics, which is a synergistic combination of probiotics and prebiotics (Roberfroid, 1998).

By targeting motility properties of bacteria in the development of probiotic cultures, Aguiar et al. (2013) selected three *B. subtilis* sp. with enhanced motility. The mixture, administered on the day of hatching, was able to reduce *C. jejuni* colonization in chicken challenged with a mixture of four different wild-type strains of *C. jejuni*. These motility-selected bacteria may have the marked ability to reach the same gastrointestinal niche in poultry, i.e., cross the protective avian intestinal mucus and reach cecal crypts, and then competitively reduce *C. jejuni*. Their findings support the theory that the motility enhancement of potential probiotic bacteria may provide a strategy for reduction of *C. jejuni* in chickens. This is also the first study to show an anti-*Campylobacter* effect of a probiotic mixture administrated once (day of hatching) on a four-strain mixture of *Campylobacter*.

### Critical parameters of *in vivo* trials

**Figure 5:**
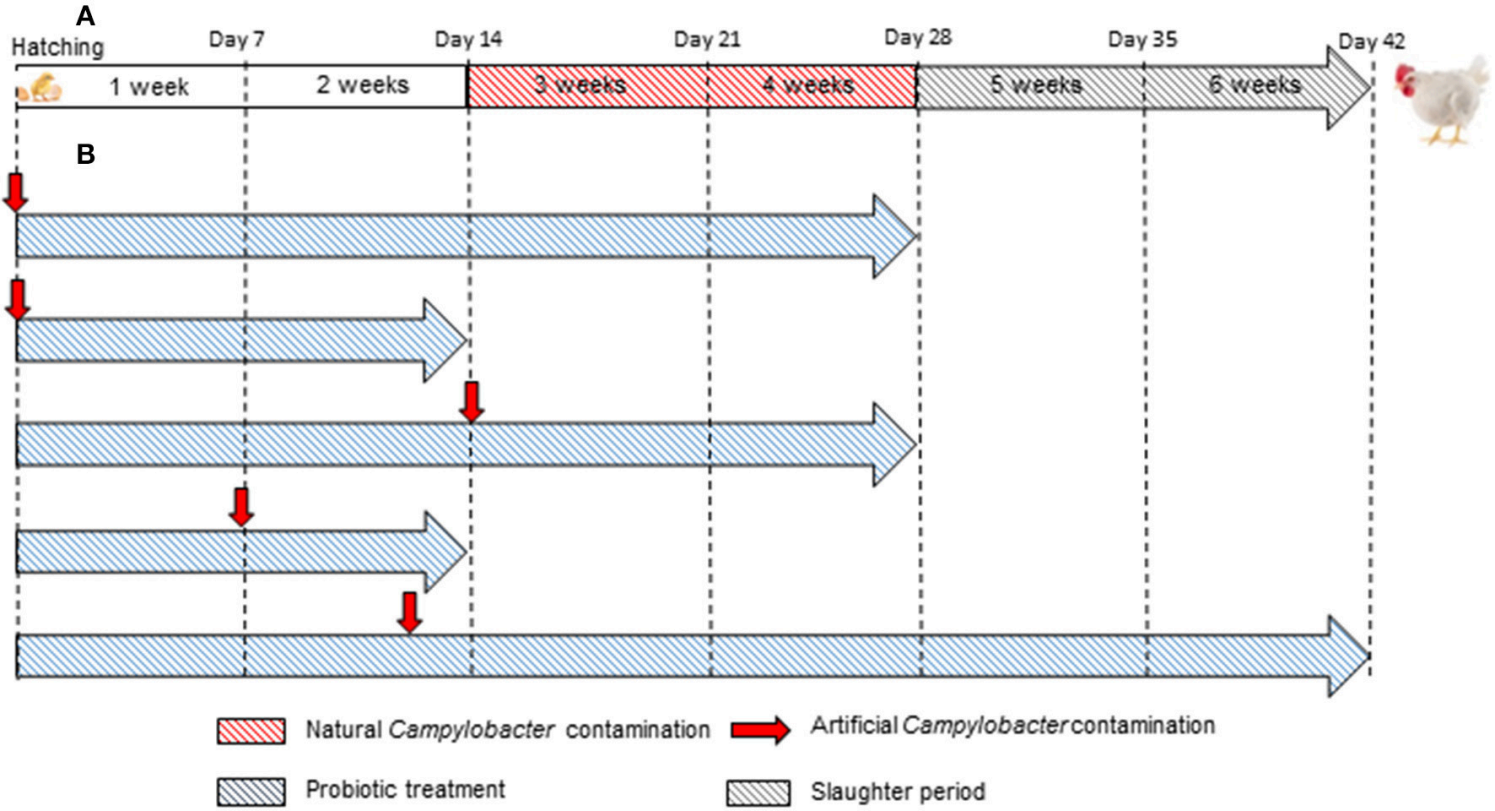
**Comparison between (A) commercial broiler chicken production and (B) *in vivo* studies designed to evaluate the efficacy of probiotics to reduce the colonization of *C. jejuni***. In almost all *in vivo* studies, the duration and/or artificial *Campylobacter* contamination are not in accordance with the duration and natural *Campylobacter* contamination in commercial broiler chicken production.

Figure 5 presents a comparison of commercial broiler chicken production and *in vivo* studies with an emphasis on the duration and timing of *Campylobacter* contamination.

Most commercial broilers reach slaughter-weight between 5 and 7 weeks of age, although slower growing races reach slaughter-weight at approximately 14 weeks of age. As indicated in Table 3 and illustrated in Figure 5, almost all *in vivo* studies have only lasted from 2 to 4 weeks. Thus, it may be very difficult to conclude about the positive or negative effectiveness of the tested probiotics at the slaughter-age of broilers. On one hand, studies may not take into account the resilience of *Campylobacter* to the presence of probiotic in the intestinal environment. The pathogen might implement strategies to overcome the anti-*Campylobacter* activity, as they do against bacteriophages (Hammerl et al., 2014) and then, despite a decrease at the beginning, the pathogen could adapt and grow once more. On the other hand, a delay could be necessary for the probiotic to express the genes required for the anti-*Campylobacter* activity, which could explain why some studies do not show positive results.

In addition, infection by *Campylobacter* is rarely detected in chicks that are less than a week old; flocks usually become infected when the birds are 2–3 weeks of age (Neill et al., 1984; Jacobs-Reitsma et al., 1995; Berndtson et al., 1996). In the majority of the studies conducted to date, researchers inoculated with *C. jejuni* in the very first days of life (Figure 5 and Table 3).

Different host factors can justify the variations in the results of probiotic use in poultry (Otutumi et al., 2012). Chicken lineage could potentially influence probiotic treatments. Recent studies showed that behavior of *C. jejuni* in the broiler chicken may differ considerably to that in chicken breeds used in experimental studies (Humphrey et al., 2015). Modern rapidly growing chicken breeds used in intensive production systems exhibited a strong inflammatory response to *C. jejuni* infection that can lead to diarrhea (Humphrey et al., 2014). As demonstrated with *Campylobacter*, probiotics could have a different efficacy depending on the chicken breeds. The immunologic status of the animals is different between different chicken breeds (Korver, 2012) and therefore is also an inherent characteristic that could modulate the probiotic action. Interactions of probiotics with different chicken breeds need to be considered. Age of the chicken when the probiotic is administrated could also affect the activity of the probiotic strain. Indeed, Mohan et al. (1996) have found that beneficial effects of probiotics on zootechnical parameters were seen during the initial growth phase, suggesting that during this stage of life the intestinal microbiota is still in an unstable condition, and the microorganisms given orally probably find a niche where they can occupy (Fuller, 1995). Therefore, the existence of an intestinal microbiota at the time of administration and the health of the host must be considered when a probiotic is supplemented for the suppression of pathogenic bacteria (Siriken et al., 2003). Antimicrobial and antiparasitic treatments received by the animals before or during the probiotic administration could also influence the survival of the probiotic strain (Jin et al., 1997).

With artificially colonized chicks, the origin of the pathogen strain is very important as the ability to colonize chickens is dependent on the original source of the isolate (Pielsticker et al., 2012). In some studies, only human isolates of *C. jejuni* (81–176, F38011) were used and might not be relevant for chicken colonization trials. As we suggest for *in vitro* assays (Sections Growth Inhibition Assays and Adhesion and Invasion Inhibition Assays), it could be interesting to include in the trials reference strains to compare results between different studies and field strains to be closer to the field.

When natural contamination occurred, it raised a particularly important point about *Campylobacter*: they exhibit high genetic and phenotypic variability (Gripp et al., 2011). As a consequence, they are not equally able to colonize chickens (Chaloner et al., 2014) and probably not equally sensitive to probiotic actions (Wine et al., 2009). Therefore, it could be important to characterize these *C. jejuni* strains. In addition, research on *Campylobacter* control has focused on *C. jejuni* and the probiotic strains used in the *in vivo* trials showed an *in vitro* anti-*Campylobacter* activity against *C. jejuni*. However, broilers can also be contaminated by *C. coli* (Rivoal et al., 2005; Hue et al., 2011) and when natural contamination occurred, *C. jejuni* could not be distinguished from *C. coli* as the enumeration was done by microbiological methods. It cannot be excluded that *C. coli* was responsible for the contamination and therefore the *in vivo* anti-*Campylobacter* activity was low or absent.

Samples used to enumerate *Campylobacter* may have an impact on the results and their interpretation. Feces and cloacal swabs are very useful for performing longitudinal studies with repeated measures on one animal. Nevertheless, cloacal swabs can only be used for detection and give information on the prevalence of *Campylobacter* while *Campylobacter* concentrations are also important when comparing an effect of a treatment. Feces could induce a bias in the results because bacterial diversity and community composition in fecal samples differ from cecal content (Pauwels et al., 2015). Bahrndorff et al. (2015) recently evaluated the colonization of individual broiler chickens by *C. jejuni* over time. They pointed out large differences between broiler chickens in the number of *C. jejuni* in cecal and fecal samples at 4, 7, and 12 days post-infection (Bahrndorff et al., 2015). These differences could be due to the fact that this foodborne pathogen requires a microaerophilic atmosphere (Macé et al., 2015) and this condition is not optimal in the fecal samples. Cecal drops could be a valuable alternative to feces and cloacal swabs in longitudinal studies (Pauwels et al., 2015).

The form and route of probiotic administration are two critical points for a future industrial application. Fresh cultures that are individually inoculated are clearly not possible at the farm level, even if the probiotics are highly active and efficient. It will be important for probiotic producers to use production processes and modified preservation and administration strategies to guarantee the delivery of active strains to the poultry. As several papers have shown, the industrial processing of a probiotic preparation has a fundamental impact on its functionality in the host (Bron et al., 2012; Van Bokhorst-Van de Veen et al., 2012). Viability, the presence or absence of pili, the cell wall condition, the matrix or the growth stage of the probiotic seem to have an important influence on its performance and its interaction with the host (Papadimitriou et al., 2015). Defining the mechanism of action of a probiotic might therefore also include some critical parameters of the production process. Their activity and survival during storage must also be assessed (FAO/WHO, 2001). Little information on these aspects are available for probiotics with an anti-*Campylobacter*. However, several studies focusing on probiotics for poultry mentioned that moisture and cell conditions have an impact on survival of probiotics during long-term storage. Freeze-drying and freezing with cryoprotective agents seemed to be suitable conditions to store probiotic strains (Pascual et al., 1999). In addition, Khoramnia et al. (2011) have shown that cryoprotectants significantly increase storage life of freeze-died lactobacilli probiotics, intended for poultry, during several months at refrigerated temperature. The probiotic could be administrated to poultry by different routes, including to animal feed. However, inclusion to the commercial feed mixture can affect probiotic survival by the temperatures used during the feed mixture storage and in the chicken incubator rooms (Pascual et al., 1999). Microencapsulation of probiotics appeared to be a promising alternative to improve their viability and survival against adverse conditions during processing, storage and gastrointestinal passage (Baffoni et al., 2012; Dianawati et al., 2015). To our knowledge, few probiotics, in the form of commercial feed additives, have exhibited a strong anti-*Campylobacter* activity (>2 log reduction) (Ghareeb et al., 2012; Guyard-Nicodème et al., 2016).

Even if the purpose of these studies is to reduce *Campylobacter* loads in poultry, it is important to keep in mind that the final destination of the broilers is the retail market. The administration of large amounts of bacteria could not only reduce *Campylobacter* but also impair the homeostasis of the avian gut microbiota. Indeed, this ecosystem is crucial for the fermentation of undigested carbohydrates (Józefiak et al., 2004). Therefore, it is necessary to examine the probiotic impact on performance parameters including average daily feed intake, body weight gain and feed conversion ratio. These parameters have not always been assessed because of the lack of a group treated with the probiotic and unchallenged with *C. jejuni* in the experimental design. In addition to zootechnical parameters, it might be interesting to monitor immune and inflammatory responses during animal experiments (Awad et al., 2014a,b; Humphrey et al., 2014).

### Impact on consumers

When considering the results of the different studies summarized in Table 3, an issue that needs to be addressed is the biological meaning of *Campylobacter* reduction in broilers. For example, the questions could be whether a statistically significant 0.5 log reduction in *C. jejuni* has important effects on the risk for consumers and what is the minimal reduction in order to conclude that a probiotic is efficient. Quantitative microbial risk assessment analyses of human campylobacteriosis associated with thermotolerant *Campylobacter* spp. in broiler chickens have been performed. In Denmark, a reduction in *Campylobacter* counts on chicken carcasses by 2 log predicted a 30-fold reduction in the incidence of campylobacteriosis in humans (Rosenquist et al., 2003). Another study conducted in Belgium demonstrated that the incidence would be reduced by 48, 85, and 96% when a 1 log, 2 log or 3 log reduction, respectively, of *Campylobacter* contamination on carcasses was achieved (Messens et al., 2007). Based on a quantitative microbiological risk assessment on *Campylobacter* in broilers at EU level, Romero-Barrios et al. (2013) estimated that the potential risk reduction would range from 48 to 100% for reductions of 1–6 log in *Campylobacter* in the intestines. According to these assessments, the minimum reduction in cecal *Campylobacter* loads that needs to be achieved to ensure a substantial reduction in human campylobacteriosis is at least 1 log_10_ CFU/g (Nauta et al., 2016).

An added value might be to extend the work to assess the impact of the probiotic strains on the prevalence or the level of *Campylobacter* on processed birds, i.e., carcasses. This could provide an overview of a part of the poultry chain production from the farm to the slaughterhouse.

## Conclusion

To conclude, research has shown that probiotics have potential for limiting *Campylobacter* colonization in broiler chickens. The oral administration of probiotic bacteria is advantageous, as they are easy to administer, i.e., in feed or drinking water, inexpensive to produce, and may persist in the animal. *In vitro* studies can indicate a possible anti-*Campylobacter* activity and yield useful information about the inhibition mechanism involved. Nevertheless, given the limitations of individual methods, no *in vitro* assay alone seems ideal to affirm a potential anti-*Campylobacter* activity. Therefore, studies must combine *in vitro* and *in vivo* methods to take into account the complexity introduced by the host, the feed, and the microbiota. This recommended combined approach may use multiple complementary tools (cell cultures, animal experiments) and address different points (molecular and overall interactions). *In vivo* studies using defined bacterial strains and various mixtures have shown promising results in reducing the colonization of *Campylobacter* spp. in chicken.

This review highlights, in particular, the intensive use of *Lactobacillus* spp., i.e., acidophilus, casei, crispatus, gasseri, helveticus, pentosus, plantarum, rhamnosus, and salivarius, which exhibit relevant *in vitro* and *in vivo* anti-*Campylobacter* activities. In the future, it may be important to investigate different and varied bacterial species.

Finally, a valuable perspective would be to look at strain combinations enhanced by prebiotics. This strategy could be relevant for additives to poultry feed for the reduction of food-borne campylobacteriosis in humans. There is still a long way to go because processing could influence the *in vivo* anti-*Campylobacter* activity.

## Author contributions

MS analyzed data from the literature and drafted the manuscript. NH, MG, and DX conceived the review, participated in its organization and helped to draft the manuscript. SM, MC, and JC revising the manuscript critically for important intellectual content. All authors read and approved the final manuscript.

### Conflict of interest statement

The authors declare that the research was conducted in the absence of any commercial or financial relationships that could be construed as a potential conflict of interest.
